# Green Synthesis and Characterization of Silver Nanoparticles Using *Spondias mombin* Extract and Their Antimicrobial Activity against Biofilm-Producing Bacteria

**DOI:** 10.3390/molecules26092681

**Published:** 2021-05-03

**Authors:** Sumitha Samuggam, Suresh V. Chinni, Prasanna Mutusamy, Subash C. B. Gopinath, Periasamy Anbu, Vijayan Venugopal, Lebaka Veeranjaneya Reddy, Balaji Enugutti

**Affiliations:** 1 Department of Biotechnology, Faculty of Applied Sciences, AIMST University, Bedong 08100, Kedah, Malaysia; sumitha@aimst.edu.my (S.S.); mutusamyprasanna@gmail.com (P.M.); 2 Institute of Nano Electronic Engineering, Faculty of Chemical Engineering Technology, Universiti Malaysia Perlis, Arau 01000, Perlis, Malaysia; subash@unimap.edu.my; 3 Department of Biological Engineering, Inha University, Incheon 402-751, Korea; anbu25@yahoo.com; 4 School of Pharmacy, Sri Balaji Vidyapeeth, Deemed to Be University, Puducherry 607402, India; vijayanv2@gmail.com; 5 Department of Microbiology, Yogi Vemana University, Kadapa 516005, India; lvereddy@gmail.com; 6 Gregor Mendel Institute (GMI), Austrian Academy of Sciences, Vienna Biocenter (VBC), Dr. Bohr-Gasse 3, 1030 Vienna, Austria; balaji.enugutti@gmi.oeaw.ac.at

**Keywords:** *Spondias mombin*, AgNP, biofilm bacteria

## Abstract

Multidrug resistant bacteria create a challenging situation for society to treat infections. Multidrug resistance (MDR) is the reason for biofilm bacteria to cause chronic infection. Plant-based nanoparticles could be an alternative solution as potential drug candidates against these MDR bacteria, as many plants are well known for their antimicrobial activity against pathogenic microorganisms. *Spondias mombin* is a traditional plant which has already been used for medicinal purposes as every part of this plant has been proven to have its own medicinal values. In this research, the *S. mombin* extract was used to synthesise AgNPs. The synthesized AgNPs were characterized and further tested for their antibacterial, reactive oxygen species and cytotoxicity properties. The characterization results showed the synthesized AgNPs to be between 8 to 50 nm with -11.52 of zeta potential value. The existence of the silver element in the AgNPs was confirmed with the peaks obtained in the EDX spectrometry. Significant antibacterial activity was observed against selected biofilm-forming pathogenic bacteria. The cytotoxicity study with *A. salina* revealed the LC50 of synthesized AgNPs was at 0.81 mg/mL. Based on the ROS quantification, it was suggested that the ROS production, due to the interaction of AgNP with different bacterial cells, causes structural changes of the cell. This proves that the synthesized AgNPs could be an effective drug against multidrug resistant bacteria.

## 1. Introduction

Nanotechnology is a recent new branch of science that has shown a wide range of development of novel technological advancements in environmental, biochemical, biological, and other applications [1]. Silver nanoparticles with the size of 1–100 nm are commonly applied in nanotechnology and science. In recent years, silver nanoparticles (AgNPs) have generated huge interest among scientists because of their impressive protection against numerous infective microorganisms. Several different ways of synthesizing AgNPs have been reported, including physical, biological, and chemical processes [2,3,4,5]. These approaches have their own benefits and drawbacks, based on their final applications. For instance, nanoparticles (NPs) synthesized through a chemical method can be immediately available for functionality testing [6]. However, chemically synthesized NPs exhibit many possible risks, including cytotoxicity, genotoxicity, carcinogenicity, and general toxicity [7,8]. On the other hand, physical methods are considered to take a longer time and are restricted to special requirements, including certain elevated temperatures or pressures, making the procedure expensive [8]. In contrast with these methods, biological methods (e.g., plant extracts, bacteria, and fungi) are known to be safe as they utilize very fewer toxic reactants or additives. This method is also considered to be rapid, simple, user-friendly, and inexpensive and includes the capability of synthesis in large quantities [9]. The synthesis of NPs using biological sources has gained interest in recent days. The application of plant extracts is highly recommended for the production of AgNPs [10]. Extracts from plant materials are high in secondary metabolites, including enzymes, polysaccharides, alkaloids, tannins, phenols, terpenoids and vitamins, which allow them to display excellent antimicrobial properties [11]. It is assumed that organic components from the leaf extract (flavonoids and terpenoids) help to stabilize the AgNPs [12].

Recently, the WHO published a list of antibiotic-resistant biofilm-producing bacteria including *Acinetobacter*, *Salmonella*, *Pseudomonas*, *Klebsiella*, *E. coli*, and *Proteus*. These bacteria cause deadly infection and are becoming resistant to most of the currently available antibiotics [13]. Multidrug resistance is the reason for biofilm-producing bacteria to contribute to chronic diseases [14]. The increasing occurrence of MDR bacteria against clinically important antibiotics has become the reason for the use of AgNPs to enhance the antibiotic effect, as AgNPs possess antibacterial, antiviral, antifungal, and also anti-inflammatory properties [15]. According to WHO, by 2050, MDR bacterial infection is predicted to kill more people than cancer and cost $100 trillion for healthcare. High research development costs and lack of profitability have long hindered the investment in novel antibiotic discovery. Consequently, using plant-based antimicrobials as an alternative therapeutic agent for the treatment of infections caused by MDR bacteria has gained popularity recently. Medicinal plants are rich in active compounds that have antimicrobial activity, and they are generally safer to use in terms of side effects, compared to conventional antibiotics. *Spondias mombin (S. mombin)* often gains attention among the researchers, due to its antimicrobial characteristics [16]. *Spondias mombin*, from the family of *Anacardiaceae* is also best-known as ambarella. This species has been used as a traditional medicine to treat diseases like anti-inflammatory and antithrombolytic complaints [17]. Every part of the *S. mombin* plant is reported to have its own medicinal values. For example, the bark of the tree is used as a treatment for diarrhea in countries like Cambodia and the fruit of *S. mombin* is used to cure itchiness, internal ulceration, sore throats, as well as skin inflammation. Evidence suggests that *S. mombin* leaves and fruits possess high antimicrobial, antioxidant, cytotoxic, antidiabetic, and thrombolytic ability [16].

In light of the importance of *S. mombin* and biologically synthesized AgNPs, the present research was designed to study plant-mediated AgNP synthesis and the characterization and predicted antimicrobial activity to counter different infective bacteria.

## 2. Results and Discussion

### 2.1. Silver Nanoparticle-Synthesis

**Figure 1:**
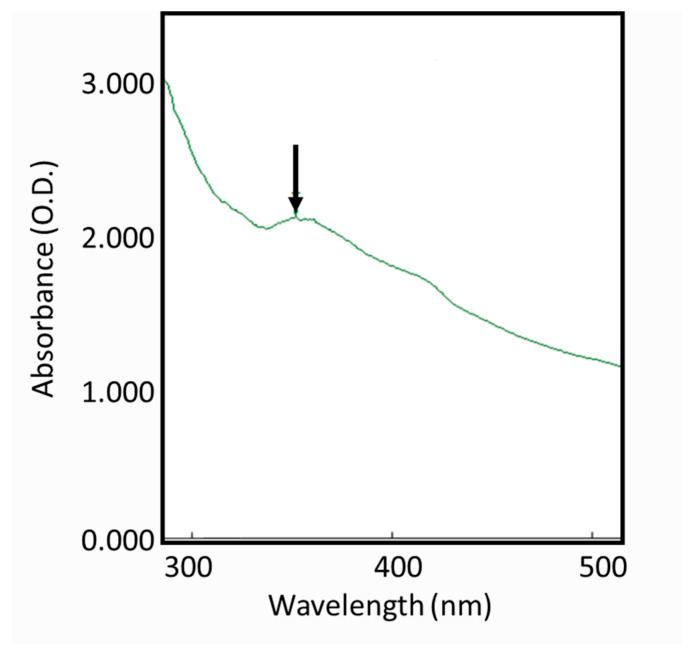
UV−Visible spectra of *Spondias mombin* leaf extract mediated silver nanoparticles.

The color change from colorless to yellowish brown suggested the reduction of AgNO_3_ had occurred by using the *S. mombin* extract as a bioreducing agent. The color change proved absorption of visible light due to the excitation of the AgNP surface plasmons (Figure 1) [18]. For further confirmation of the reduction of Ag ions to AgNPs, the maximum absorbance of UV−VIS spectra of different wavelengths was obtained (Figure 1). It was observed that there was no peak obtained in the silver nitrate (AgNO_3_) as it did not contain any reducing agent. However, the AgNO_3_ with the plant extract showed maximum intensity between the wavelengths of 300 to 400 nm, confirming the role of *S. mombin* leaf extract as a reducing agent to form AgNPs.

A study showed that in the biosynthesis of AgNPs by the reduction of Ag ions, the terpenoids in the leaf extract play a crucial role [19]. A previous study reported this function of terpenoids from *Geranium* leaves in the biosynthesis of AgNPs [20]. A similar process might have worked in the current study, where the flavonoids and phenolic compounds from *S. mombin* extract acted as a capping and stabilizing agent in the formation of NPs.

### 2.2. Characterization of Synthesized Silver Nanoparticles

**Figure 2:**
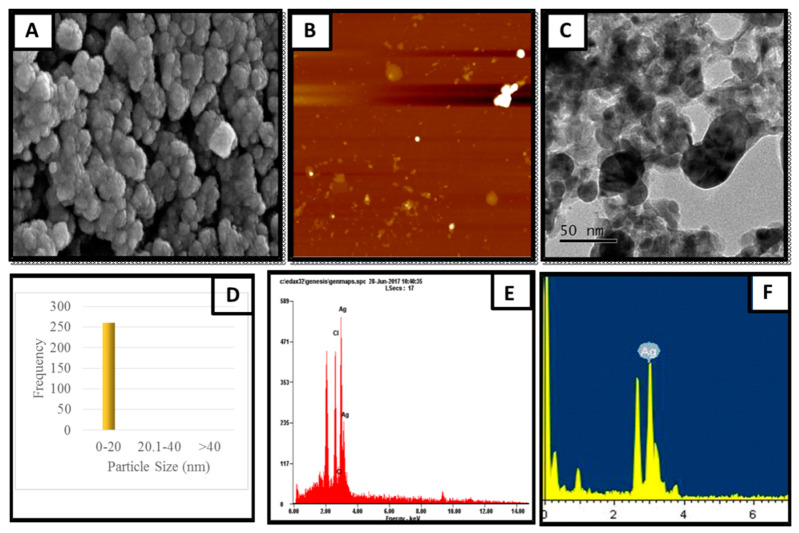
Structural characteristics of produced AgNPs. (**A**) SEM image with the scale of 200 nm; (**B**) spherical AgNPs observed using AFM; (**C**) TEM image with the scale of 50 nm; (**D**) histogram representing AgNP size distribution; (**E**) energy dispersive X-ray spectroscopy analysis with field emission scanning electron microscopy; (**F**) energy dispersive X-ray spectroscopy analysis with TEM.

The particle size of the nanoparticle is an important consideration in biological applications and it strongly affects the diffusion rate via biological membranes. Previous reports showed that the smaller the size of the nanoparticle, the higher its permeability, but showed increased toxicity. Thus, a suitable size is highly recommended for specific biological functions. Hence, scanning electron microscopy (SEM) was used to study the surface morphology. Figure 2 clearly shows that the AgNPs were spherical in shape with smooth edges. The mean particle size of AgNPs was 17 nm, which is the appropriate size (8–50 nm) for biological membrane permeation, and this is the tolerable range for inducing toxicity within cells.

**Figure 3:**
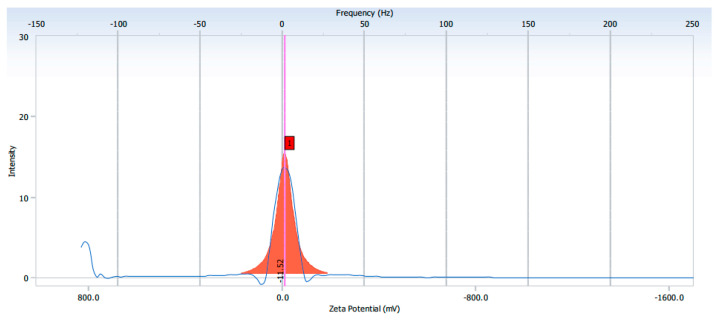
The zeta potential value of synthesized AgNPs.

**Figure 4:**
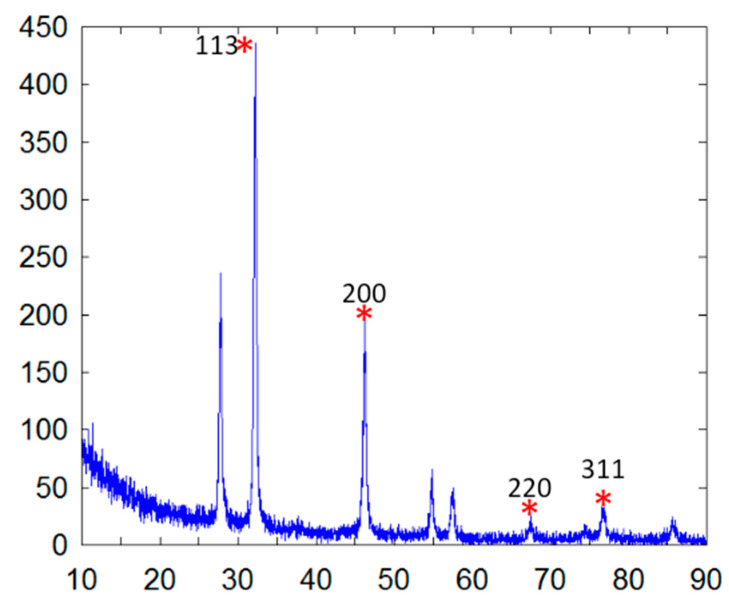
X-ray diffraction pattern of synthesized AgNPs. Ag peaks are marked (*) and 2θ values are given.

The elemental composition was revealed by EDX analysis of the synthesized AgNPs. EDX analysis was also used to determine the amount of each element in the formation of AgNPs. Based on Figure 2E,F, two peaks were observed in the spectrum in between 2 to 4 keV. The peak that formed at 3 keV showed the existence of an elemental Ag signal, as AgNPs have optical peaks at ~3 keV due to major emission energies. Another peak indicated the presence of elemental chlorine which could have been from the plant extract. The zeta potential was found to be −11.52 mV for the synthesized AgNPs from the *S. mombin* leaf extract (Figure 3). Based on the zeta potential value of the AgNPs, it was shown the AgNPs had moderate stability. This could be due to the existence of bioactive contents in the extract. Thus, the particles might aggregate and flocculate due to the absence of a repulsive force. It was also observed that there was the presence of some weak peaks at 28°, 54°, 57° and 86° which might have been from the organic compound in the leaf extract (Figure 4) [21,22].

### 2.3. Antibacterial Activity of Spondias Mombin Leaf Extract

The antibacterial activity of *S. mombin* ethanolic leaf extract for selected bacteria was studied. A ciprofloxacin commercial antibiotic disc was used as positive control whereas the 10% DMSO was employed as negative control.

**Figure 5:**
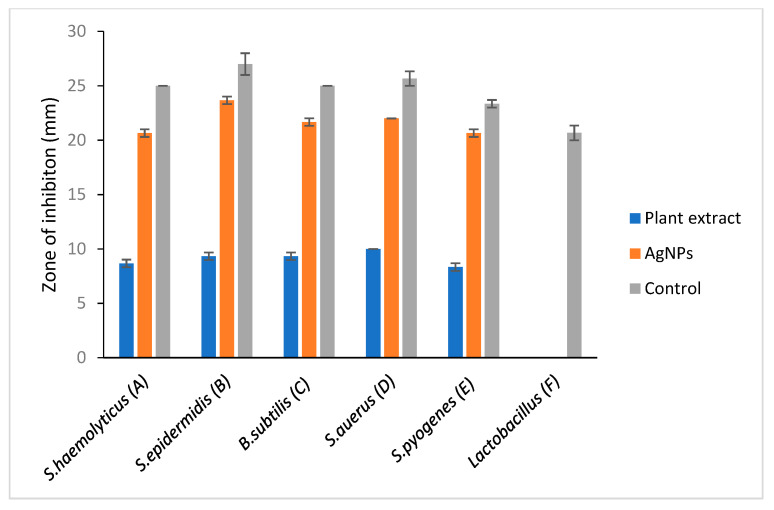
Antimicrobial analysis against selected Gram-positive bacteria. A: *Staphylococcus haemolyticus*, B: *Staphylococcus epidermidis*, C: *Bacillus subtilis*, D: *Staphylococcus aureus*, E: *Streptococcus pyogens*, F: *Lactobacillus*. Comparative evaluation of selected Gram-positive bacteria vs. zone of inhibition.

**Figure 6:**
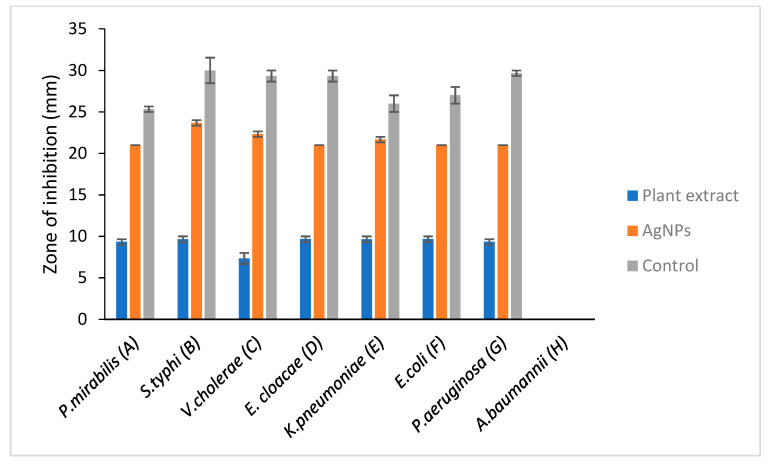
Antimicrobial activity against selected Gram-negative bacteria. A: Proteus mirabilis, B: Salmonella typhi, C: Vibrio cholera, D: Enterobacter cloacae, E: Klebsiella pneumoniae, F: E. coli, G: Pseudomonas aeruginosa, H: Acinetobacter baumannii. Comparative evaluation of selected Gram-negative bacteria vs. zone of inhibition.

A clear zone of inhibition indicates a deterrent to the bacteria from growing. Results that were obtained from this study showed that *S. mombin* leaf extract had its own antimicrobial activity as they produced a clear zone of inhibition against the bacteria tested. From the results obtained, the nanoparticles showed an equal level of antimicrobial activity towards *Enterobacter cloacae*, *Escherichia coli, Klebsiella pneumoniae* and *Salmonella typhi*. Besides that, *Vibrio cholera* showed the lowest zone of inhibition compared to the other bacteria (Figure 5 and Figure 6). However, there was not any zone of inhibition observed when the plant extract was tested with *Lactobacillus*. This showed that the plant extract had no antimicrobial property against *Lactobacillus*. As the bacteria have proven health benefits, this *Lactobacillus* group are classified as ‘generally recognized as safe’ bacteria [23]. They are categorized as nonpathogenic bacteria and the most common type of lactic acid bacteria in food and feed products [24]. The resistance of *Lactobacillus* towards *S. mombin* leaf extract could serve as an alternative treatment against human pathogenic bacteria as it does not affect the normal human flora population.

*Acinetobacter baumannii* also showed no zone of inhibition for positive control which was the ciprofloxacin commercial antibiotic disc. This resistance of *A. baumannii* is mainly due to the mutation in the quinolone resistance determining region of DNA gyrase [25].

The known antimicrobial mechanism of the plant extract against various bacteria was inhibiting the cell wall synthesis, accumulating in the bacterial membrane which caused energy depletion or interference with the permeability of the cell membrane. This would eventually result in mutation, cell damage and the death of the bacteria. There is a study reporting that the phenolic and flavonoid content in the plant extract is the reason for the immune-modulator organs killing the bacteria [26].

### 2.4. Antibacterial Activity of Synthesized Silver Nanoparticles

The antimicrobial activity of silver nanoparticles (AgNPs) synthesis from *S. mombin* leaf extract using ethanol as solvent with disc diffusion method is tabulated in Table 1 and Table 2. For the positive and negative control, ciprofloxacin commercial antibiotic disc and 10% DMSO were used, respectively.

A significant antimicrobial activity showed in the presence of *S. mombin* capped AgNPs against the selected bacteria. Silver nanoparticles synthesized from *S. mombin* leaf extract showed high antimicrobial activity for *Staphylococcus epidermidis* and *Salmonella typhi*. *Proteus mirabilis*, *Enterobacter cloacae*, *Escherichia coli*, *Pseudomonas aeruginosa* and showed a constitutive level of antimicrobial activity against the synthesized silver nanoparticle. Since there was no antibacterial activity observed in the plant extract against *Lactobacillus* and *Acinetobacter baumanii*, these strains were not tested with synthesized AgNPs.

When AgNPs contact with moisture, Ag^+^ ions are released. The Ag^+^ ions react with nucleic acid mainly with nucleosides forming the complex of the bacteria. AgNPs accumulate and form something called a ‘pit’ in the bacteria’s cell wall and the nanoparticles slowly penetrate the intracellular component of the bacteria. The silver particles cause the plasma membrane to detach from the cell wall. This results in a loss of DNA replication and the protein synthesis process is also inhibited which causes the death of the bacteria. In addition to that, the hindrance of biofilm formation by AgNPs is an important mechanism, as biofilm plays a crucial part in the development of bacterial resistance against common drugs [27].

AgNPs demonstrated mediocre antibacterial action in Gram-positive bacteria compared to Gram-negative bacteria, depending on the result. This is due to the Gram-positive bacteria having a thick peptidoglycan layer. This causes difficulty in spreading AgNPs across the cell wall to disrupt the cell’s activity and to inhibit its growth [28]. Gram-positive bacteria are made up of 70–100 peptidoglycans layers. Peptidoglycan consists of two polysaccharides, N-acetyl-glucosamine and N-acetyl-muramic acid, interlinked with peptide side chains and cross bridges [29]. On the other hand, compared to Gram-negative bacteria, the outer membrane of Gram-positive bacteria might cause less silver to reach the cytoplasmic membrane [30]. As a result, Gram-positive bacteria displayed a higher tolerance to synthesized silver nanoparticles relative to Gram-negative bacteria.

**Figure 7:**
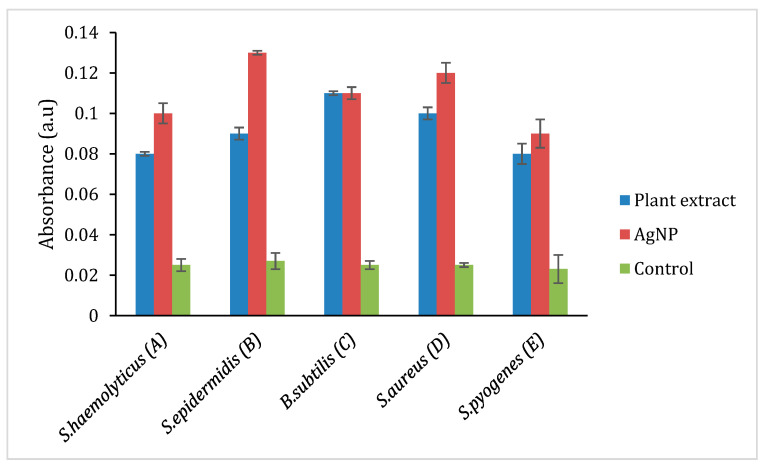
ROS production in selected Gram-positive bacteria. A: Staphylococcus haemolyticus, B: Staphylococcus epidermidis, C: Bacillus subtilis, D: Staphylococcus aureus, E: Streptococcus pyogenes.

**Figure 8:**
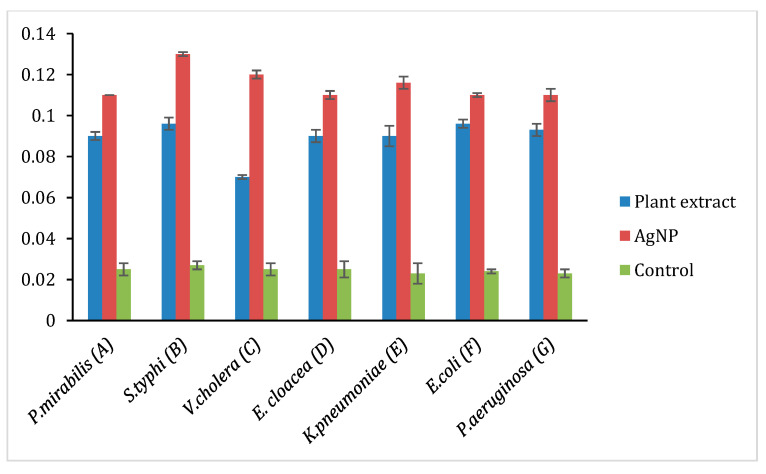
ROS production in selected Gram-negative bacteria A: Proteus mirabilis, B: *Salmonella typhi*, C: *Vibrio cholera*, D: *Enterobacter cloacae*, E: *Klebsiella pneumoniae*, F: *E. coli*, G: *Pseudomonas aeruginosa*.

The oxidation of AgNP releases Ag^+^ ions, and the ions are responsible for circulation in the living organism. The production of reactive oxygen species (ROS) induces oxidative stress that damages the membrane, proteins, DNA/RNA, and lipids, which enhance the cytotoxicity in prokaryotic cells. Thus, the ROS production was analyzed in these selected Gram-positive (*S. haemolyticus*, *S. epidermidis*, *B. subtilis*, *S. aureus*, *S. pyogenes*) and Gram-negative bacterial strains (*P. mirabilis*, *V. cholera, K. pneumoniae*, *E. coli*, *P. aeruginosa*, *E. cloacae, S. typhi.*) by treating with plant extract, AgNP and ciprofloxacin to quantify the amount of ROS production in contrast to the negative control (DMSO), and the findings are represented in Figure 7 and Figure 8. However, Figure 7 shows that the ROS level in Gram-positive bacteria and plant extract showed its effect in the following order. i.e., *S. aureus, S. epidermidis, B. subtilis, S. haemolyticus,* and *S. pyogenes*. AgNP showed an excellent ROS level in all the strains as compared with ciprofloxacin. Figure 8 shows the ROS production level in the Gram-negative strains and the plant extract showed a similar level in all the strains. AgNP showed significant ROS level in *S. typhi* and *V. cholera* followed by other strains as compared with ciprofloxacin. Based on the results, it is suggested the ROS production is due to the interaction of AgNP with different bacterial cells. The interaction causes structural changes of the cell by causing toxicity by inducing oxidative stress. This affects the protein synthesis process resulting in cell death.

### 2.5. Cytotoxic Study

**Figure 9:**
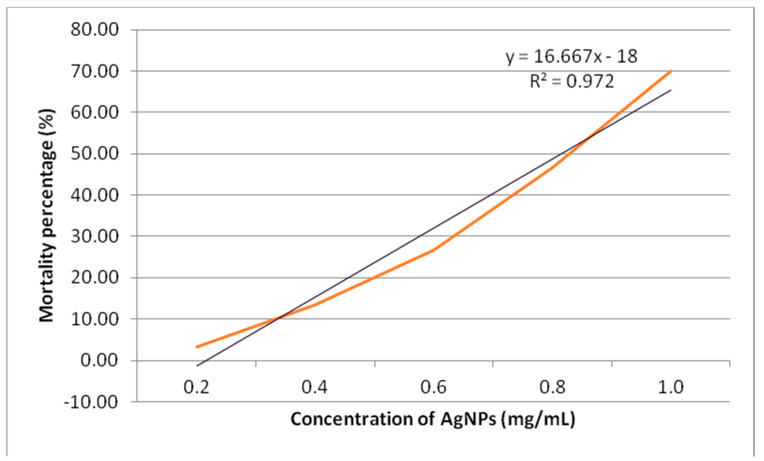
The cytotoxicity rate of synthesized AgNPs using *A. salina.*

The cytotoxicity study revealed that the highest mortality of 70% was obtained at 1.0 mg/mL. Figure 9 shows the plot of mortality percentage against the various concentrations of synthesized AgNPs. The graph showed a direct proportional relationship between the concentration of synthesized AgNPs and the rate of mortality. The LC_50_ of synthesized AgNPs was observed to be at 0.81 mg/mL. A study was conducted by Samuggam et al. using *Durio zibethinus* AgNPs showed the LC_50_ was 3.03 mg/mL [28]. Another study conducted by Shriniwas et al., reported the LC_50_ value of AgNPs synthesized using L. *camara* L. was 0.51 mg/mL [29]. The *A. salina* cytotoxicity depends on the AgNP size. It was stated that the cytotoxicity activity would be stronger when the size of the AgNPs was smaller [30].

## 3. Materials and Methods

### 3.1. Collecting the Plant Samples

Healthy, disease free young leaves of *S. mombin* were accumulated from Kulim, Kedah. These samples were shade dried for 2 weeks and crushed into powder form. The fine powdered leaves were stored in an airtight container at room temperature until they were required.

### 3.2. Preparation of S. mombin Leaf Extract

Fifty grams of powdered plant materials and 250 mL of solvent (99.98% ethanol) were added to a conical flask. This conical flask was further positioned in the incubator at 180 rpm at 37 °C for 7 days. Then, the extracted components of the plant were filtered and concentrated at temperatures around 35 °C–40 °C with the help of a rotary evaporator. The concentrated extract was air dried and the leaf extract was stored at 4 °C.

### 3.3. Biosynthesis of Silver Nanoparticles

AgNPs were biosynthesized according to the method mentioned previously (15–17). A millimolar solution of silver nitrate was prepared. The mixture was mixed with the magnetic mixer until it fully dissolved the silver nitrate crystals. The AgNO_3_ solution was applied with five milliliters of plant extract slowly, until the hue shifted from pale yellow to brown. For 19 h in the dark room, the solution was incubated. After 19 h, the solution was centrifuged, for 15 min at 4000 rpm and the supernatant discarded. The pellet was then cleaned, scattered, and poured out into the glass of the clock with purified water. The pellet was air dried and stored for further use at 4 °C.

### 3.4. Characterization of Synthesized Silver Nanoparticles

To detect the reduction of the aqueous silver ion by scanning from 300 to 900 nm to obtain the maximum absorption strength of AgNPs, the UV−visible spectrophotometer (Beckman Coulter DU 800 Spectrophotometer, Williamston, SC, USA) was used. Scanning electron microscopy (SEM) and transmission electron microscopy (TEM) were used to analyze the morphological and structural features of the synthesized AgNPs. SEM and TEM analysis were performed on a Hitachi, S-4300 SE, Japan and a JEM-2100F, JEOL, Japan, respectively. The EDX analysis was performed to study the elemental composition of the AgNPs. Both SEM and TEM were equipped with energy-dispersive X-ray analysis. AgNP FESEM photographs were taken under a high-energy electron beam with a working distance of 15 kV and 4.5 mm. Surface texture analysis was carried using atomic force microscopy (AFM) using Nano Scope, Ica, Vecco, Plainsview, NY, USA. The sample for AFM analysis was conducted by preparing a thin pellet of AgNPs on a glass slide which could dry for 5 min. Crystalline nature of AgNP was determined using an X-ray Diffractometer (DMAX-2500, Rigaku, Tokyo, Japan). The diffraction angle was varied from 10° to 90° at 40 kV and 100 mA with Cu Ka radiation source. The size distribution and stability of AgNPs were studied using particle size analyzer (PHOTAL OTSUKA ELECTRONICS, ELC-Z model, Osaka, Japan).

### 3.5. Verification of the Antibacterial Activity of Synthesized Silver Nanoparticles

Kirby−Bauer antibiotic test (disc diffusion test) was used in this project. In this test, 10 bacteria were used as the testing microorganisms which consisted of Gram positive and Gram negative. They were *Bacillus subtilis*, *Escherichia coli*, *Staphylococcus aureus*, *Enterobacter cloacae*, *Staphylococcus epidermidis*, *Klebsiella pneumoniae*, *Vibrio cholera*, *Salmonella typhi*, *Staphylococcus haemolyticus*, *Proteus mirabilis*, *Streptococcus pyogenes*, *Pseudomonas aeruginosa*, *Lactobacillus* and *Acinetobacter baumannii*. The antimicrobial activity between the AgNPs and leaf extract was analyzed by the zone of inhibition formed on the agar plates.

### 3.6. Reactive Oxygen Species (ROS) Quantification

The Choi et al., 2006 method was used to determine the amount of reactive oxidative species (ROS) released by the microbes. To conclude, a total of 200 mL of bacterial strain was applied with 1 mL of plant extract, AgNP, ciprofloxacin (positive control) and DMSO (negative control) and kept in 37 °C incubator shaker. Once 6 h of incubation had been achieved, the bacteria suspension was centrifuged at 11,000× *g* for 11 min at low temperature to obtain the pellet. The pellet was applied with 2% Nitro Blue Tetrazolium (NBT) mixture. This pellet was kept at room temperature for 60 min in dark conditions. After centrifugation of the solution, the supernatant was removed, and the pellet was rinsed twice using PBS before another centrifugation at 9000× *g* for 3 min. The obtained pellet containing cells membrane was disrupted by treating with 2 M KOH solution. A sample of 50% DMSO was combined with the solution and followed by 10 min incubation at room temperature to dissolve formazan crystals. The solution was again centrifuged and 100 μL of the supernatant was distributed to 96 well plates. The absorbance was calculated at 620 nm using ELISA reader.

### 3.7. Cytotoxicity Study

The cytotoxicity study was done using *Artemia salina* (*A. salina*) according to Samuggam et al. [28]. Total of 10 larvae of *A. salina* were incubated at different concentrations of AgNPs in range of 0.2 to 1.0 mg/mL in 1 milliliter of sterilized seawater. This *A. salina* was incubated for 16 h and 8 h of light and dark, respectively, at 25 °C for 24 h. The assay was carried out in triplicate. Based on the larval mortality percentage, the LC_50_ values were determined.

## 4. Conclusions

In conclusion, *Spondias mombin* mediated silver nanoparticles proved their antibacterial ability against biofilm-producing bacteria, *S. haemolyticus*, *S. epidermidis*, *B. subtilis*, *S. aureus*, *S. pyogenes, Enterobacter cloacae*, *Escherichia coli, Klebsiella pneumoniae* and *Salmonella typhi*. The property of antibacterial activity of this plant extract was improved by synthesizing *Spondias mombin* leaf extract with capped silver nanoparticles. The production of ROS and cytotoxicity studies suggested the interaction of AgNPs with the bacterial cells caused structural changes which led to cell death and less cytotoxicity, respectively. Thus, these synthesized silver nanoparticles have the potential to be an effective drug against biofilm-producing bacteria.

## Figures and Tables

**Table 1:** Antimicrobial activity of ethanolic extract of *S. mombin* and AgNP produced with ethanolic extract of *S. mombin* counter to Gram-positive bacteria. The information is presented in the table with the mean (±SE, standard error), *p* < 0.05.

Gram-Positive Bacteria	Zone of Inhibition (mm)			
	Plant Extract (Ethanolic)	Silver Nanoparticles (AgNPs)	Controls	
			Positive (Ciprofloxacin)	Negative
*S.* *haemolyticus*	8.67 ± 0.35	20.65 ± 0.35	25	-
*S. epidermis*	9.35 ± 0.35	23.65 ± 0.35	27 ± 1.00	-
*B.* *subtilis*	9.35 ± 0.35	21.65 ± 0.35	25	-
*S. aurus*	10	22	25.67 ± 0.67	-
*S. pyogenes*	8.33 ± 0.35	20.65 ± 0.35	23.35 ± 0.35	-
*Lactobacillus*	0.00	-	20.67 ± 0.67	-

**Table 2:** Antimicrobial activity of ethanolic extract of *S. mombin* and AgNP synthesized with ethanolic extract of *S. mombin* against Gram-negative bacteria. The information presented in the table with the mean (±SE, standard error), *p* < 0.05.

Gram-Negative Bacteria	Zone of Inhibition (mm)			
	Plant Extract (Ethanolic)	Silver Nanoparticles (AgNPs)	Controls	
			Positive (Ciprofloxacin)	Negative
*P. mirabilis*	9.33 ± 0.33	21	25.33 ± 0.33	-
*S. typhi*	9.65 ± 0.35	23.67 ± 0.33	30 ± 1.53	-
*V. cholera*	7.33 ± 0.67	22.33 ± 0.33	± 0.67	-
*E. cloacae*	9.67 ± 0.33	21	± 0.67	-
*K. pneumoniae*	9.65 ± 0.35	0.33	26 ± 1.00	-
*E. coli*	0.33	21	26 ± 1.00	-
*P.* *aeruginosa*	9.33 ± 0.33	21	29.7 ± 0.33	-
*A. baumannii*	0.00	-	-	-

## Data Availability

Data is contained within the article.

## Sample Availability

Samples of the compounds or not available from the authors.
